# Development and Application of nanoPCR Method for Detection of Feline Panleukopenia Virus

**DOI:** 10.3390/vetsci10070440

**Published:** 2023-07-06

**Authors:** Haowen Xue, Yang Liang, Xu Gao, Yanhao Song, Kunru Zhu, Meng Yang, Jingrui Hao, Haoyuan Ma, Kai Yu

**Affiliations:** 1 Laboratory for Animal Molecular Virology, Department of Veterinary Medicine, College of Agricultural, Yanbian University, Yanji 133002, China; xhw704601416@foxmail.com (H.X.); syh20230516@163.com (Y.S.); zkr13596766195@163.com (K.Z.); y2439082877@outlook.com (M.Y.); m17808001838@163.com (J.H.); bakougenn@outlook.com (H.M.); 13044365811@163.com (K.Y.); 2 Beijing Shengzetang Animal Hospital, Beijing 102218, China; m1678621548@163.com

**Keywords:** feline panleukopenia virus, VP2 gene, nanoPCR, clinical testing, epidemiology

## Abstract

**Simple Summary:**

Feline panleukopenia is a severe infectious disease caused by the feline panleukopenia virus. It has caused great obstacles to the breeding of pet cats and the protection of rare feline animals such as lions and tigers. There is currently no effective therapy for FPV infection. The virus is extensively dispersed in the environment, and infected animals expel the virus in their feces, allowing the virus to spread; young kittens are especially vulnerable. Infection in kittens frequently is acute, and some cats may die unexpectedly without any signs, posing a significant challenge to early clinical identification. Some of the existing detection methods, such as qPCR, immunohistochemical analysis, colloidal gold test strips, etc., are time-consuming, laborious, costly, and difficult to operate, which is not conducive to popularization. Although colloidal gold test strips are fast and convenient, they have low sensitivity. A nanoPCR reaction was successfully created in this work by adding gold nanoparticles to the traditional PCR reaction system, and was capable of detecting FPV infection in the clinical situation.

**Abstract:**

Feline panleukopenia (FP) is a severe viral illness caused by the feline panleukopenia virus (FPV), putting sectors like companion cat breeding and endangered feline conservation at risk. The virus has a high morbidity and fatality rate and is found all over the world. We created a novel FPV assay using nanoPCR technology and assessed the method’s specificity and sensitivity. The approach amplified a 345 bp nucleic acid fragment with a minimum detection limit of 7.97 × 10^2^ copies/μL, which is about 100 times greater than traditional PCR. We collected anal swabs from 83 cats suspected of FPV infection for practical application, and the FPV-positive rate determined by the nanoPCR approach was 77.1%. In conclusion, the approach is more sensitive than conventional PCR and more convenient and cost-effective than qPCR methodology and may be utilized for the clinical detection of FPV.

## 1. Introduction

Feline panleukopenia (FP) is an acute, highly contagious infection caused by the feline panleukopenia virus (FPV) [1], which can spread rapidly in cats. FPV was first discovered in 1928 and officially named in 1935 [2,3]. FPV produces severe fever, vomiting, and diarrhea in infected animals, as well as a large drop in white blood cells; it is highly lethal in young cats. The virus spreads quickly and can be transmitted horizontally by infected animals’ excrement, urine, and vomit and vertically during pregnancy to the fetus, where it can cause fetal cerebellar hypoplasia [4].

FPV, commonly known as feline distemper virus, is a single-stranded DNA virus of the family Parvoviridae and the genus parvovirus that exhibits a round or hexagonal form under an electron microscope with no capsule structure [5]. Its genome is around 5200 nt in length and contains two open reading frames (ORFs) that encode structural proteins (VP1 and VP2) and non-structural proteins (NS1 and NS2) [6,7]. The NS protein in FPV mainly regulates the expression and replication of viral genes after viral infection, while the VP2 protein is the translation product of the VP2 gene and the main component of the viral capsid. It is highly conserved and stable, and is the main immune protective antigen protein of FPV [8]. The VP2 proteins of different parvoviruses have great differences in host cell specificity and monoclonal antibody recognition, so they are often selected as target genes for genetic engineering, and VP2 genes are also used for related identification. After the viral genome and capsid have been assembled, a portion of the VP2 protein is cleaved to produce a new VP3 protein [9].

Mullis et al. devised the polymerase chain reaction (PCR) technology in 1985 [10]. This technology duplicates the process of in vitro DNA synthesis through enzymatic reaction, may amplify the target gene fragment exponentially, and has the benefits of high sensitivity, good specificity, cheap cost, and fast speed, making it extensively employed in pathogen detection. In less than 40 years since 1985, advanced methods such as nested PCR [11], multiplex PCR [12], real-time PCR (qPCR) [13], droplet digital PCR (ddPCR) [14], nanoPCR [15], and insulated isothermal PCR (iiPCR) [16] have been invented for clinical testing based on following the principles of the technique.

With the development of materials science, various nanoparticle materials have penetrated the biological and medical industries. For example, nano-silver is added to drugs [17] or wound dressings [18] because of its excellent antibacterial ability [19]. Nano-zinc can be added to feed as a nutrient [20], which can play a role in growth promotion and immune regulation [21]. NanoPCR is a relatively recent PCR method. The process involves adding gold nanoparticles with diameters smaller than 100 nm to the PCR reaction. During the reaction temperature shift, such particles will form nanofluid, enhancing the thermal conductivity of the reaction and allowing it to quickly reach the target reaction temperature in the thermal cycle, reducing the reaction time spent at a non-target temperature [22]. It also greatly enhances the kinetic contact of reactant components through charge effects, etc., thus improving the pairing efficiency of primers and templates [23]. Gold nanoparticles can also bind to the Taq enzyme reversibly, and the combination of the two is closer at lower temperatures, which can inhibit non-specific amplification. At higher temperatures, the combination of the two is loose and can perform normal specific amplification. This material also has a role similar to the single-stranded binding protein (SSB), which can selectively adsorb single-stranded DNA without adsorbing double-stranded DNA, improving the signal-to-noise ratio of PCR reaction [24]. This further improves the efficiency, specificity, and sensitivity of PCR reactions, which can be 10–1000 times more sensitive than conventional PCR and are less costly, easier to operate, and do not require specialized instruments as required by highly sensitive qPCR methods.

The commonly used colloidal gold test paper procedure for FPV infection is fast but ineffective. Existing detection methods rely on viral isolation and serological identification, but they are time-consuming and costly, making them unsuitable for rapid detection of clinical samples. With the rapid growth of the pet cat industry, existing FPV detection systems have faced considerable challenges. Based on epidemic surveillance, prevention, and control of cats, it is proposed to create a low-cost, simple, and more sensitive detection approach for the early diagnosis and monitoring of FPV that does not require the instrumentation of qPCR.

## 2. Materials and Methods

### 2.1. Virus and Clinical Samples

FPV YBYJ-1 strain was isolated and preserved by our laboratory in advance, and feline herpesvirus (FHV-1), feline caliciviruses (FCV), and feline coronavirus (FCoV) were preserved by our laboratory. A total of 83 cat anal swabs (Table S1) suspected to be infected with FPV were collected from multiple animal clinics in Jilin Province. Among them, 47 were positive for FPV by colloidal gold test strips (Anigen, Korea), and the rest were negative for FPV. After soaking swab samples in 0.9% saline solution, the leachate was centrifuged at 5000× *g* for 3 min to obtain the supernatant. The materials above were extracted with a viral DNA/RNA extraction kit (CWBIO, Taizhou, China) and kept at −80 °C.

### 2.2. Primer Design

The FPV VP2 gene sequence was retrieved from GenBank, and primers to amplify the full-length VP2 gene of FPV were constructed using Oligo 7 software (Molecular Biology Insights, Amsterdam, Netherlands): VP2-F: 5′-ATGAGTGATGGAGCAGTTCAAC-3′ and VP2-R: 5′- GTATACCATATAACAAACCTTC-3′, with a predicted amplification length of 1932 bp. For the construction of standard positive template plasmids, specific primers were designed according to the conserved region of the VP2 gene: VP2-U: 5′-TATATAGCACATCAAGATACG-3′ and VP2-L: 5′-TGCATCAGGATCATATTCATT-3′, the expected amplified fragment length was 345 bp. Specific primers were designed for SYBR Green I real-time PCR: VP2-S1: 5′- GTGATGGAGCAGTTCAACCAG -3′ and VP2-S2: 5′- CCCGTTCCCAGATCCTGTAG -3′, the expected amplified fragment length was 74 bp.

### 2.3. Establishment of PCR Reaction

The VP2 gene was amplified by VP2-F/R primer. The reaction conditions were 95 °C for 5 min, 94 °C for 30 s, 55 °C for 2 min, 72 °C for 2 min, a total of 30 cycles, and 72 °C for 5 min. Electrophoresis analysis was performed using 1% agarose gel. The target band with a length of 1932 bp was recovered and purified using a gel recovery kit, containing the pMD19-T vector (Takara, Kyoto, Japan) and propagated in DH5α (TransGen, Beijing, China). After expanded culture, the recombinant pMD19-T-FPV-VP2 plasmid was extracted using a plasmid mini kit (Omega, CT, USA), and the concentration and copy number (7.97 × 10^10^ copies/μL) were determined using Nanodrop (Thermo Fisher Scientific Inc., MA, USA) and used as a standard positive template. The VP2-U/L primer was used to amplify the standard positive template by PCR. The reaction system was plasmid template 1 μL, 1 μL of upstream and downstream primers (10 μM), 2 μL of dNTPs (2.5 mM each), 2.5 μL of 10 × Ex Taq Buffer (20 mM), 0.5 μL of Ex Taq DNA polymerase (5 U/μL) (Takara, Japan), dissolved in ddH_2_O, total volume 25 μL. The PCR reaction procedure was 95 °C 5 min, 95 °C 30 s, 50 °C 30 s, 72 °C 30 s, a total of 30 reaction cycles, 72 °C 10 min, and finally stored at 4 °C. At the same time, the nanoPCR experimental group was set up. Compared with the above conventional PCR reaction system, 1 μL of gold nanoparticles (diameter 20 nm, concentration 0.2 mM) was added to the total volume of 25 μL, and the reaction was carried out according to the same reaction conditions. The above reactions were carried out in the PCR instrument (NYX Technik Inc., San Diego, CA, USA), and finally, 8 μL of reaction products were analyzed by 1% agarose gel electrophoresis.

### 2.4. Optimization of Reaction System and Procedure for nanoPCR

The particle diameter and concentration of the gold nanoparticles utilized in the reaction system were modified. The diameter of gold nanoparticles (Jieyi, Shanghai, China) in each reaction system was varied between 10 and 40 nm. The number of particles supplied was varied between 0.5 μL (0.1 mM) and 2.5 μL (0.5 mM).

A gradient of 0.2 μL (0.1 μM) to 2 μL (1.0 μM) was used to control the amount of primer used. The best annealing temperature of the primers was determined, and an annealing temperature gradient of 45 to 55 °C was established.

1% agarose gel electrophoresis was used to analyze the PCR products.

### 2.5. Sensitivity Detection

To compare the sensitivity of conventional PCR methods with nanoPCR methods using optimal reaction conditions, serial tenfold dilutions of standard positive templates were used, and ddH_2_O was used as the negative control instead of the template. Reaction products were analyzed by 1% agarose gel electrophoresis.

### 2.6. Specificity Testing

FHV and FPV DNA, as well as FCoV and FCV RNA, were isolated, and cDNA was generated using a reverse transcription kit (Takara, Kyoto, Japan). Negative controls were from anal swabs from cats that had not been infected with FPV, and reaction products were examined using 1% agarose gel electrophoresis.

### 2.7. Initial Clinical Application

A total of 83 anal swabs from sick cats suspected of FPV infection were collected from several animal hospitals in Jilin Province, and some of them had tested positive for FPV using colloidal gold test paper. The swabs were processed and all swabs were tested simultaneously by the nano-PCR method, the conventional PCR method and the qPCR method.

### 2.8. Analysis of the Genetic Evolution of Amino Acids

Selected samples that tested positive for FPV by nanoPCR were sent to Kumei Biotechnology Co., Ltd. (Changchun, China) for sequencing of the VP2 gene. The returned DNA sequences were translated into amino acids, and a genetic evolutionary tree was constructed for analysis based on the published sequences in GenBank. The evolutionary tree was constructed using MEGA X software (Mega Limited, Auckland, New Zealand) with 1000 replicate determinations using the Neighbor-Joining (N-J) method.

## 3. Results

### 3.1. Establishment of nanoPCR Method and Reaction Optimization

**Figure 1:**
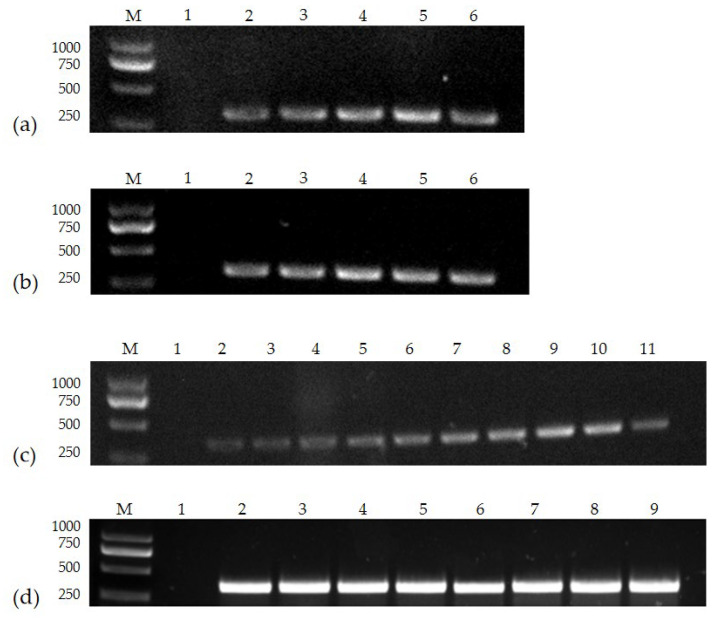
Optimization of particle diameter, (**a**) particle concentration, (**b**) primer concentration, and (**c**) annealing temperature (**d**): (**a**) Lane M, DL 2000 Marker; Lane 1, negative control; Lanes 2–6, 10, 15, 20, 30, and 40 nm; (**b**) Lane M, DL 2000 Marker; Lane 1, negative control; Lanes 2–6, 0.1, 0.2, 0.3, 0.4, and 0.5 mM; (**c**) Lane M, DL 2000 Marker; Lane 1, negative control; Lanes 2–11, 0.1, 0.2, 0.3, 0.4, 0.5, 0.6, 0.7, 0.8, 0.9, and 1.0 μM; (**d**) Lane M, DL 2000 Marker; Lane 1, negative control; Lanes 2–9, 55.0, 54.2, 52.9, 51.0, 48.6, 46.9, 45.7, and 45.0 °C (please find the WB full membrane in Figure S1).

The number of primers used, the diameter of the nanoparticles, and the amount of particles added were all tested in the nanoPCR buffer system. The results showed that when the particle diameter was 30 nm, the particle addition amount was 1.5 μL (0.3 mM), the primer addition amount was 1.6 μL (0.8 μM), and the annealing temperature was 52.9 °C, detection of the correct electrophoretic band (345 bp) was optimal (Figure 1).

### 3.2. Sensitivity of the nanoPCR Method

**Figure 2:**
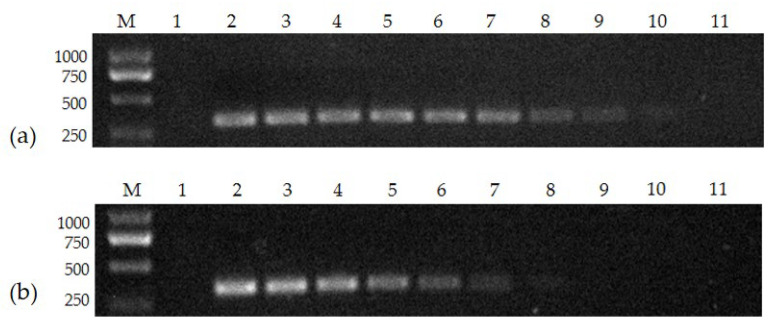
The sensitivity of nanoPCR (**a**) and conventional PCR (**b**): (**a**) Lane M, DL 2000 Marker; Lane 1, negative control; Lanes 2–11, 7.97 × 10^10^, 7.97 × 10^9^, 7.97 × 10^8^, 7.97 × 10^7^, 7.97 × 10^6^, 7.97 × 10^5^, 7.97 × 10^4^, 7.97 × 10^3^, 7.97 × 10^2^, and 7.97 × 10^1^ copies/μL; (**b**) Lane M, DL 2000 Marker; Lane 1, negative control; Lanes 2–11, 7.97 × 10^10^, 7.97 × 10^9^, 7.97 × 10^8^, 7.97 × 10^7^, 7.97 × 10^6^, 7.97 × 10^5^, 7.97 × 10^4^, 7.97 × 10^3^, 7.97 × 10^2^, and 7.97 × 10^1^ copies/μL (please find the WB full membrane in Figure S1).

The FPV-VP2 standard positive template was constructed at a concentration of 7.97 × 10^10^ copies/μL, serially diluted tenfold to 7.97 × 10^1^ copies/μL, and amplified using both the conventional PCR method and the nanoPCR. The electrophoresis results showed that the minimum detection limit of the nanoPCR method was 7.97 × 10^2^ copies/μL, which was about 100 times higher than that of the conventional PCR (7.97 × 10^4^ copies/μL) (Figure 2).

### 3.3. Specificity of the nanoPCR Method

**Figure 3:**
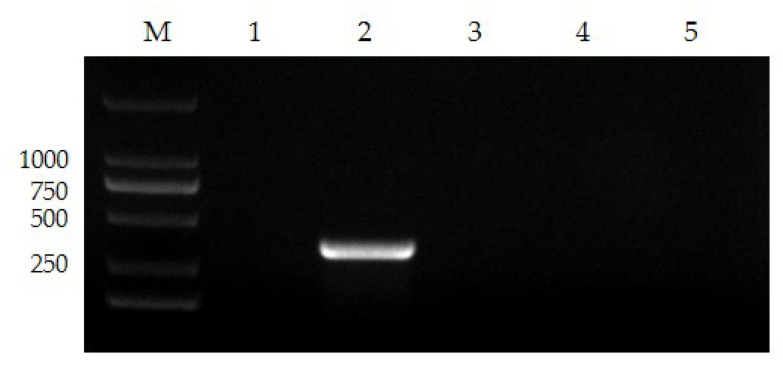
The specificity of nanoPCR. Lane M, DL 2000 Marker; Lane 1, negative control; Lanes 2–5, FPV, FHV, FCoV, and FCV (please find the WB full membrane in Figure S1).

The nanoPCR approach was shown to be precise enough to properly amplify FPV genes in the identification of distinct viruses from the same host, whereas the genes of other viruses could not be amplified (Figure 3).

### 3.4. Initial Clinical Application of the nanoPCR Method

**Figure 4:**
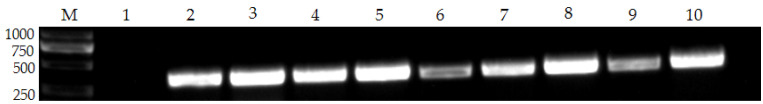
The detection results of some FPV clinical samples. Lane M, DL 2000 Marker; Lane 1, negative control; Lanes 2–10, FPV clinical samples (please find the WB full membrane in Figure S1).

Based on the adjusted reaction conditions, 83 samples were evaluated by nanoPCR and conventional PCR at the same time (Figure 4). The nanoPCR method recognized 64 samples as positive for FPV (77.1%, 64/83) and the conventional PCR detected 51 samples as positive for FPV (61.4%, 51/83). There were no situations where the conventional PCR indicated positive samples and the nanoPCR was negative. This suggests that the nanoPCR approach is more sensitive than the conventional PCR for FPV clinical testing. In addition, after sensitivity testing, qPCR can achieve a minimum detection limit of 7.97 × 10^1^ copies/uL, which is 10 times that of the nanoPCR method established in this study. In the detection of clinical samples, the positive results of FPV detected by qPCR were consistent with the results of nanoPCR (Table S1).

### 3.5. Genetic Evolutionary Analysis of Some Positive Samples

**Figure 5:**
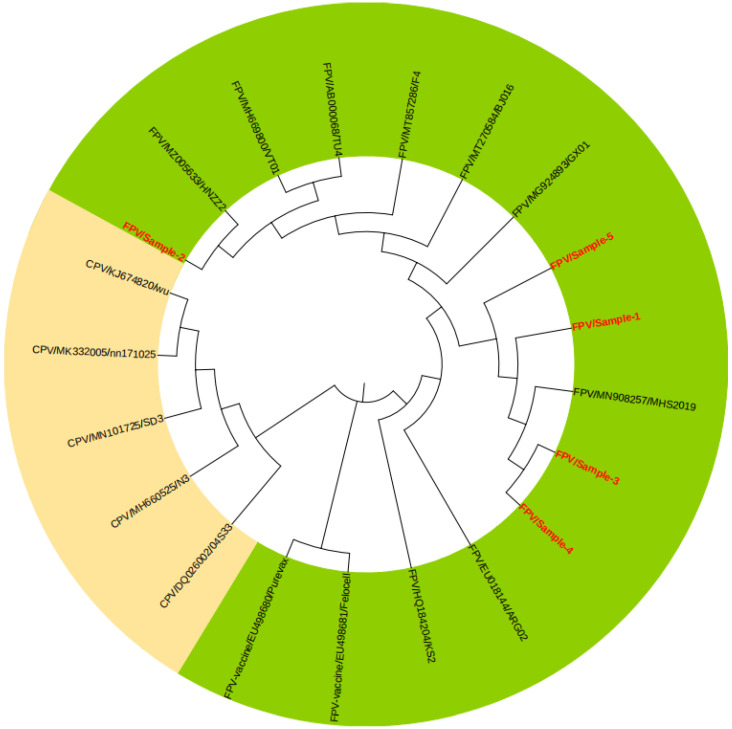
Phylogenetic analysis of FPV with CPV based on the VP2 amino acid sequence by the Neighbor-Joining (N-J) method. The green background is the FPV branch, and the yellow background is the CPV branch. The red fonts represent some of the clinical samples in this study.

The amino acid sequence of the VP2 gene was obtained by sequencing several clinically identified FPV-positive samples. A total of 21 strains of FPV and canine parvovirus (CPV) registered in GenBank were selected to construct a phylogenetic tree (Figure 5). The results showed that the two parvoviruses constituted two different branches, and the positive strains from this test were closely related to MHS2019, GX01, BJ016, and HNZZ2 from China and were all in the FPV branch, which was far from CPV, indicating that the local FPV strains had not yet evolved towards CPV. However, the two vaccine strains (EU498680/Purevax and EU498681/Felocell) were not on the same branch in the phylogenetic tree as the sample strains, suggesting a large genetic difference between them, which could be one of the reasons for the reduced vaccine protection.

## 4. Discussion

FPV was first discovered by Verge and Cristoforoni at the end of the 1920s in the last century [25], and it was first successfully isolated from the clouded leopard in 1964 by Bilin and Johnson [2]. In recent years, there has been an increase in the number of cases related to this virus infection [26,27]. Globally, FPV outbreaks and research are being published, and the virus has had a significant influence on pet cat breeding and rare feline conservation businesses. Despite the widespread use of commercially available vaccinations, FPV infection continues to kill a huge number of felines. FPV induces diarrhea in infected animals, and so a large number of virus particles are diffused in the environment, which can quickly spread infection, putting a burden on current clinical detection procedures. Due to the high rate of infection and mortality of this disease, the danger, and the lack of very effective treatment, there is an urgent need for a rapid, sensitive, simple, and low-cost test for the detection and prevention of FPV which can be widely promoted.

Previous traditional laboratory diagnostic methods for the detection of FPV relied on pathogenic, serological, and molecular biological methods. The first successful isolation of FPV in primary feline kidney cell cultures was performed by Johnson et al. [2] in 1964; O’Reilly et al. [28] went on to develop a cat embryonic cell line for subsequent FPV isolation and study in 1969. However, the procedure of isolating and identifying viruses using cell cultures is too time-consuming and demanding for the experimental setting to be acceptable for early clinical testing. PCR technology has been constantly refined for diagnostic purposes since its introduction [29]. In 1995, Schunck et al. [30] pioneered the amplification and identification of FPV using touch-down PCR techniques with success. This technology replaces the earlier method of directly finding and seeing viral particles with an electron microscope, considerably boosting detection efficiency, accuracy, and convenience. Due to its extraordinarily high sensitivity, qPCR began to be employed in clinical testing with the advancement of PCR technology. In recent years, many researchers have developed a large number of methods for FPV detection using TaqMan probes [31,32] and SYBR Green I fluorescent dye methods [33,34], all of which have considerable sensitivity and specificity, and the minimum detection limit can reach 10^1^ copies/μL [34]. The minimum detection limit of nanoPCR in this study was 7.97 × 10^2^ copies/μL, which was slightly lower than that determined by SYBR Green qPCR which was determined to be 7.97 × 10^1^ copies/μL on the sample. However, the instrumentation, reagents, and operating stringency required for qPCR technology are difficult to support at grassroots levels of clinical diagnostics. Although the sensitivity of nanoPCR is slightly lower than that of qPCR, with the development of materials science, the screening and optimization of materials such as nanoparticles can be continued in the future, so that nanoPCR has better detection ability. In comparison to other FPV detection methods, this study intends to improve the traditional PCR method through incorporating gold nanoparticles into the traditional PCR reaction system, relying on the excellent thermal conductivity of gold particles to develop a highly sensitive and specific FPV nanoPCR detection method. The method has the advantages of being low-cost, having a simple and fast operation, having no special instrument, and being suitable for large-scale promotion.

Currently, nanoPCR technology has been applied to the detection of various pathogens such as bovine respiratory syncytial virus (BRSV) [35], mink enteritis virus (MEV) [36], and canine coronavirus (CCV) [37], etc. VP2 gene of FPV is highly conserved and the translated VP2 protein is able to stimulate the body’s immune system to produce neutralizing antibodies. The VP2 protein is the principal antigenic protein of FPV; it determines the virus’s hemagglutination activity and plays a role in the viral infection of host cells [38]. As a result, the VP2 gene was chosen as the target gene in this work, a standard positive plasmid was generated, serially diluted tenfold, and nanoPCR amplification was conducted. The results showed that the nanoPCR method could specifically detect the presence of FPV, and the sensitivity of the detection could reach 100 times that of the conventional PCR method, indicating that the nanoPCR assay developed in this study could be used to detect early FPV infection.

Living conditions of human populations are steadily improving, the pet industry is developing quickly, and people’s desire for pet cats is increasing, which means that an epidemic of FPV infection might have a significant economic impact. The nanoPCR method was used to detect FPV in 83 samples collected from June 2022 to January 2023 in various animal hospitals in Jilin Province; 64 FPV-positive samples were detected by the nanoPCR method, with a detection rate of 77.1% (64/83), and 51 FPV-positive samples were detected by the conventional PCR method, with a detection rate of 61.4% (51/83). We found that nanoPCR can be applied to the clinical detection of early FPV infection with results consistent with qPCR, and has the advantages of low cost and easy operation.

After comparing the VP2 amino acid sequences of some FPV-positive samples, it was found that the other four samples except Sample 2 had A91S mutations, and other positions were conserved, with only a small number of scattered mutations. Our group conducted an epidemiological investigation of FPV in the Yanbian area of Jilin Province in the early stage, and the results were similar to the comparison results of this sample [39]. There were no key mutations, indicating that the local FPV strains were very conservative and did not have the trend of evolution to CPV, which was consistent with N. Decaro et al. [40]. The evolution of FPV derived from a total of 39 strains from the United Kingdom and Italy was at a standstill. Compared with the two vaccine strains, the 232nd amino acid of the two vaccine strains was I, which was consistent with CPV. In the genetic evolution analysis, the branch of the vaccine strain was far from the branch of the sample, which may be the reason why some cats were still infected with FPV after vaccination.

We evaluated the conserved sequences in our work by comparing the FPV genome sequences and created appropriate primers for use in nanoPCR. We discovered that the optimal amount of nanogold particle addition for this method is 0.3 mM, the optimal particle diameter is 30 nm, and the minimum detection limit is 7.97 × 10^2^ copies/μL, which is approximately 100 times more sensitive than conventional PCR. Due to its excellent sensitivity and specificity, the nanoPCR method can be used for early clinical low-titer infection, and can also be used for a wide range of epidemiological studies to achieve the purpose of monitoring virus variation.

## 5. Conclusions

This study developed a nanoPCR detection method for FPV detection, which has better sensitivity than colloidal gold test strip and traditional PCR. The minimum detection limit is 7.97 × 10^2^ copies/μL, which has sufficient specificity and can be used for the early clinical detection of FPV.

## Data Availability

The data that supports the findings of this study are available in the supplementary material of this article.

## Supplementary Materials

The following supporting information can be downloaded at https://www.mdpi.com/article/10.3390/vetsci10070440/s1, Figure S1: WB full membrane for Figure 1, Figure 2, Figure 3 and Figure 4; Table S1: Symptoms and vaccination of 83 samples.
