# Supplementary material for: Development and Application of nanoPCR Method for Detection of Feline Panleukopenia Virus
Source: Vet Sci. 2023 Jul 6;10(7):440. doi: 10.3390/vetsci10070440 (PMC10386105; doi:10.3390/vetsci10070440)
Supplement: Supplementary file 1 [file vetsci-10-00440-s001.zip › Figure S1.pdf]

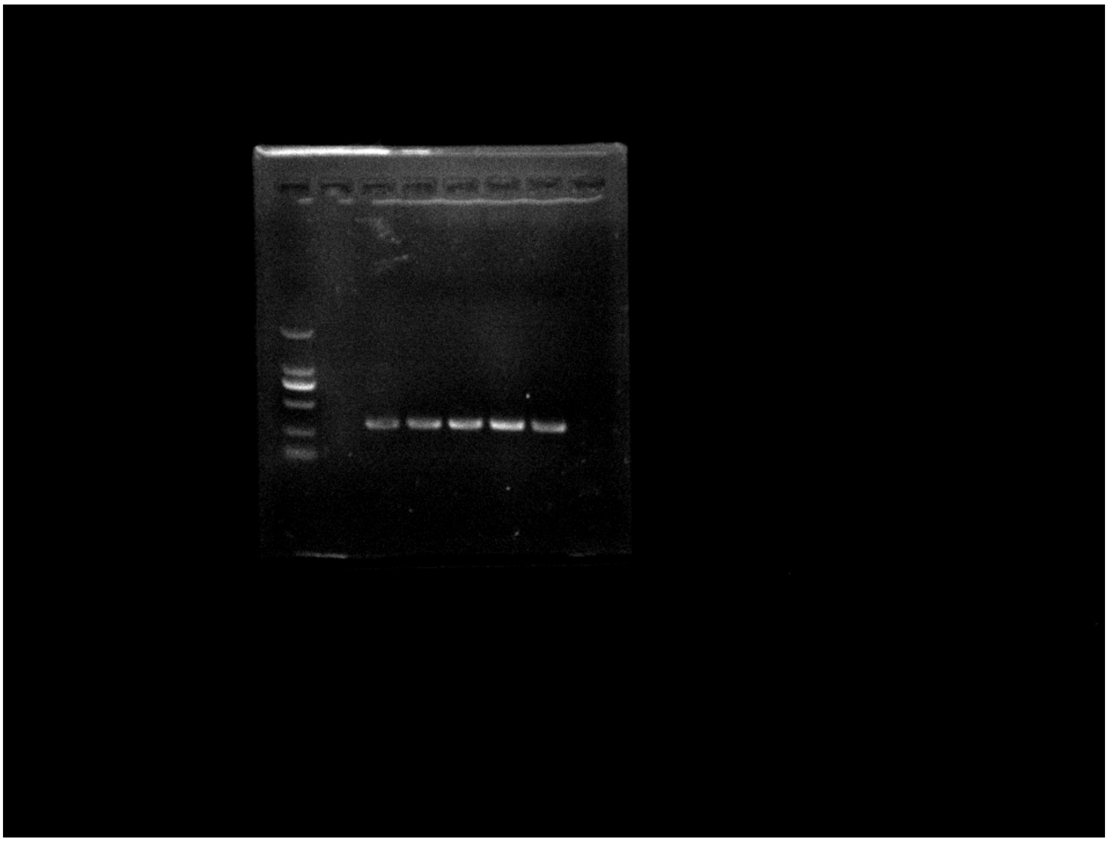

Figure 1(a). particle diameter (a) Lane M, DL 2,000 Marker; Lane 1, negative control; Lanes 2-6, 10, 15, 20, 30 and 40nm.

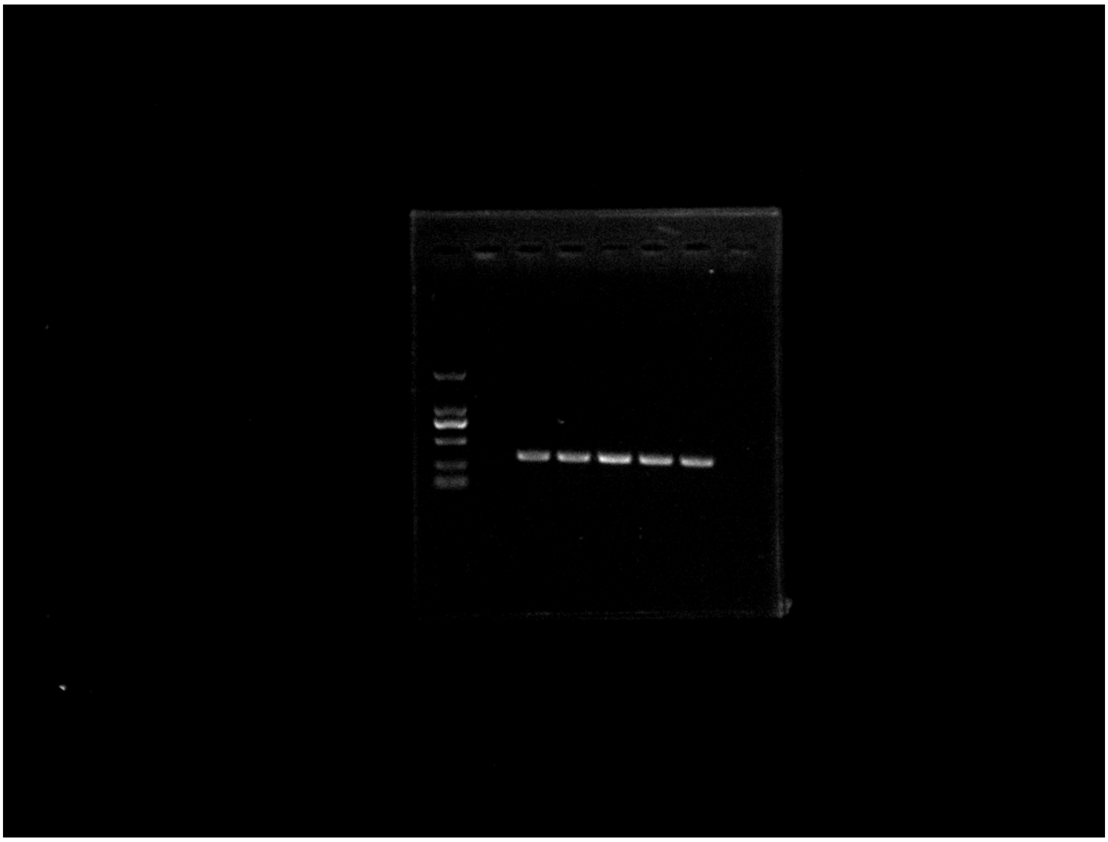

Figure 1(b). particle concentration (b) Lane M, DL 2,000 Marker; Lane 1, negative control; Lanes 2-6, 0.1, 0.2, 0.3, 0.4 and 0.5 mM.

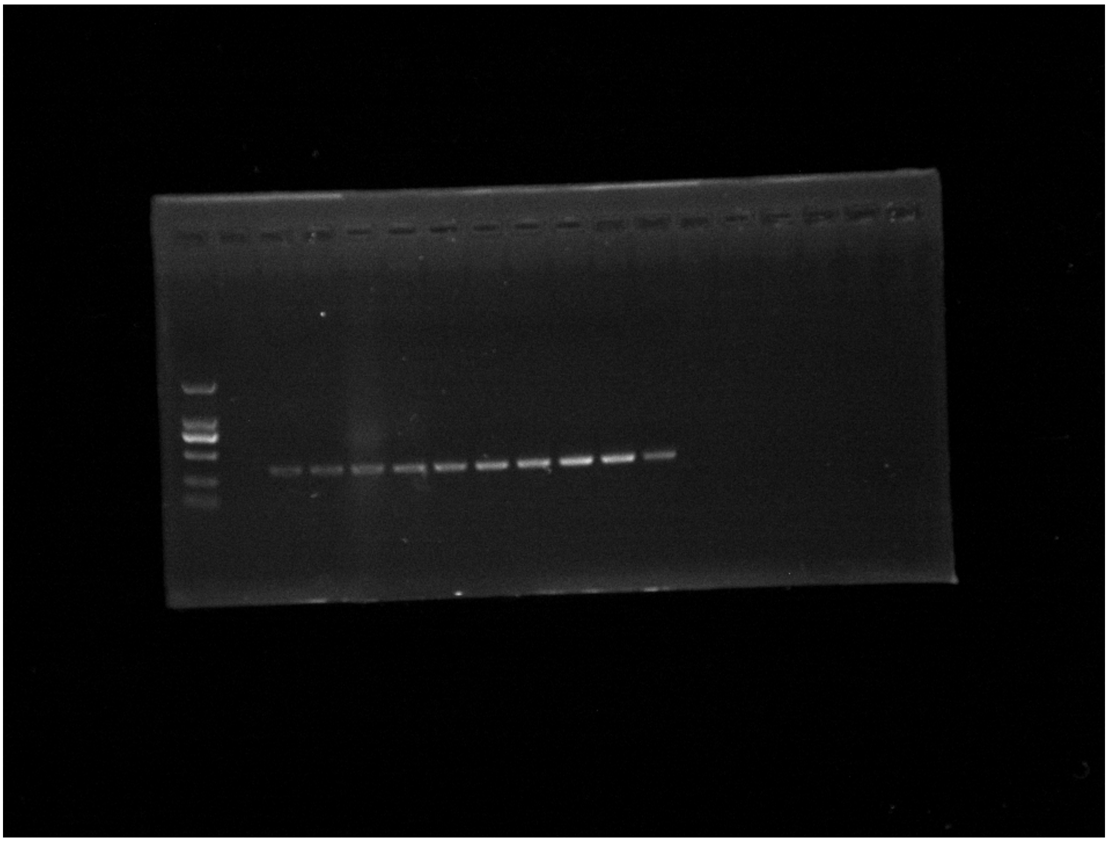

Figure 1(c). Optimization of primer concentration (c) Lane M, DL 2,000 Marker; Lane 1, negative control; Lanes 2-11, 0.1, 0.2, 0.3, 0.4, 0.5, 0.6, 0.7, 0.8, 0.9, 1.0μM.

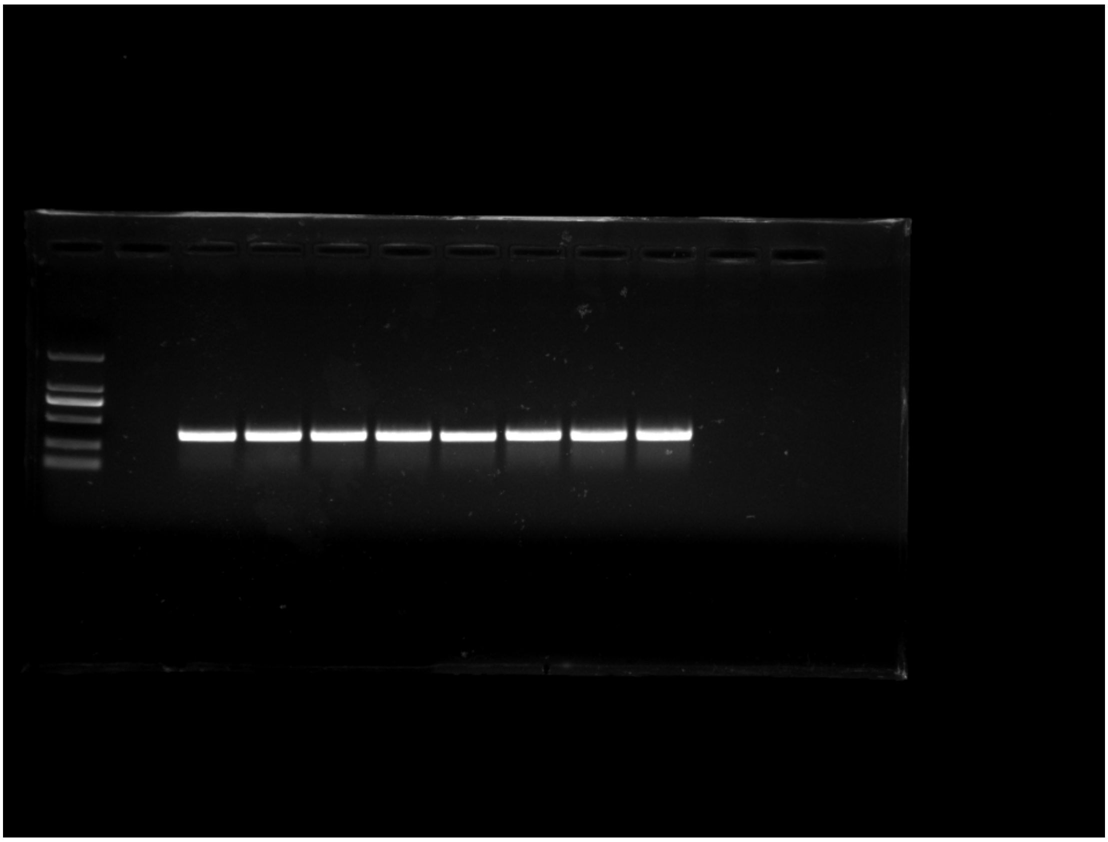

Figure 1(d). annealing temperature (d) Lane M, DL 2,000 Marker; Lane 1, negative control; Lanes 2-9, 55.0, 54.2, 52.9, 51.0, 48.6, 46.9, 45.7 and 45.0 °C.

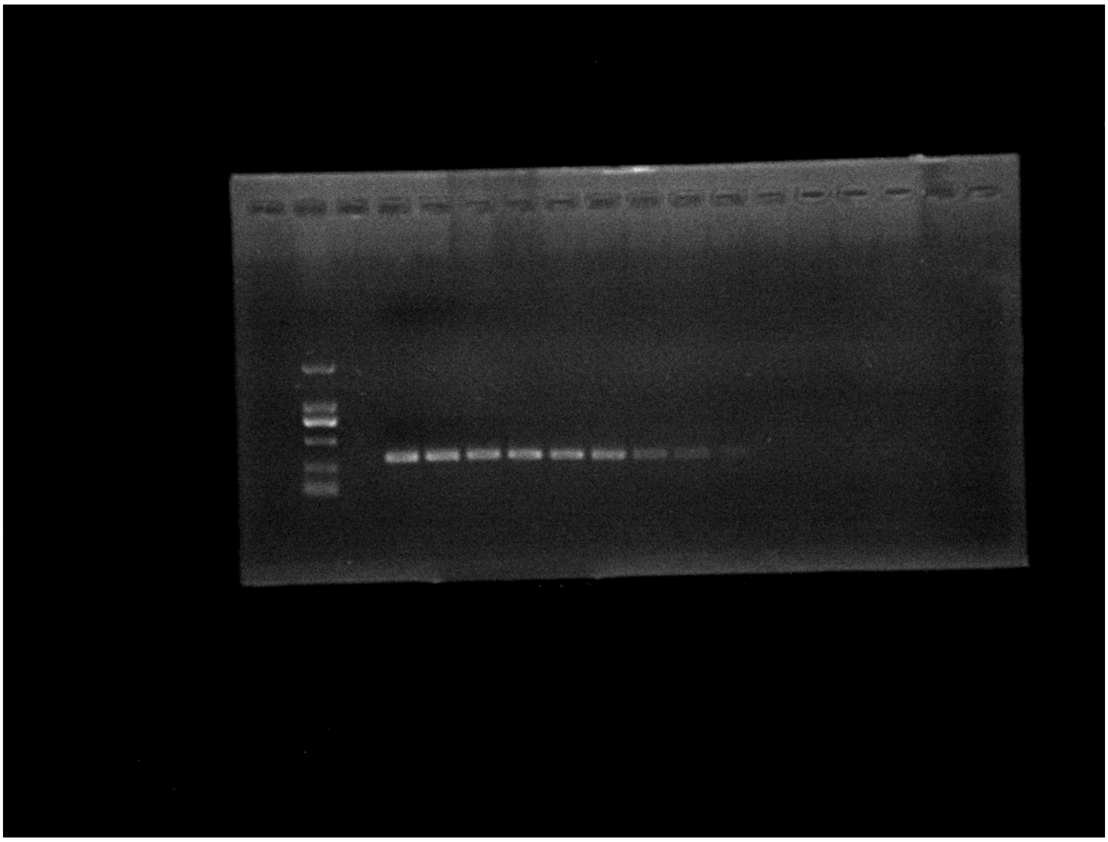

Figure 2(a). The sensitivity of nanoPCR (a) Lane M, DL 2,000 Marker; Lane 1, negative control; Lanes 2-11, 7.97×10^{10}, 7.97×10^{9}, 7.97×10^{8}, 7.97×10^{7}, 7.97×10^{6}, 7.97×10^{5}, 7.97×10^{4}, 7.97×10^{3}, 7.97×10^{2} and 7.97×10^{1} copies/μL.

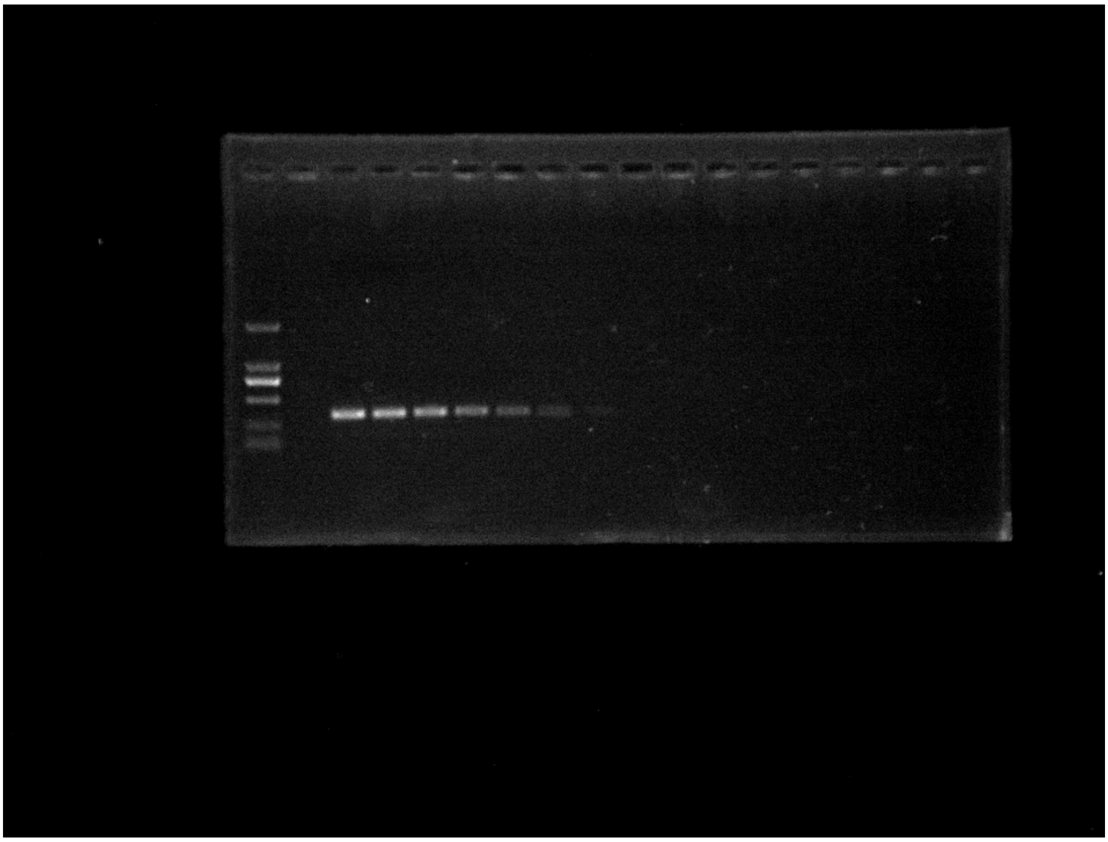

Figure 2(b). The sensitivity of conventional PCR (b) Lane M, DL 2,000 Marker; Lane 1, negative control; Lanes 2-11, 7.97×10^{10}, 7.97×10^{9}, 7.97×10^{8}, 7.97×10^{7}, 7.97×10^{6}, 7.97×10^{5}, 7.97×10^{4}, 7.97×10^{3}, 7.97×10^{2} and 7.97×10^{1} copies/μL.

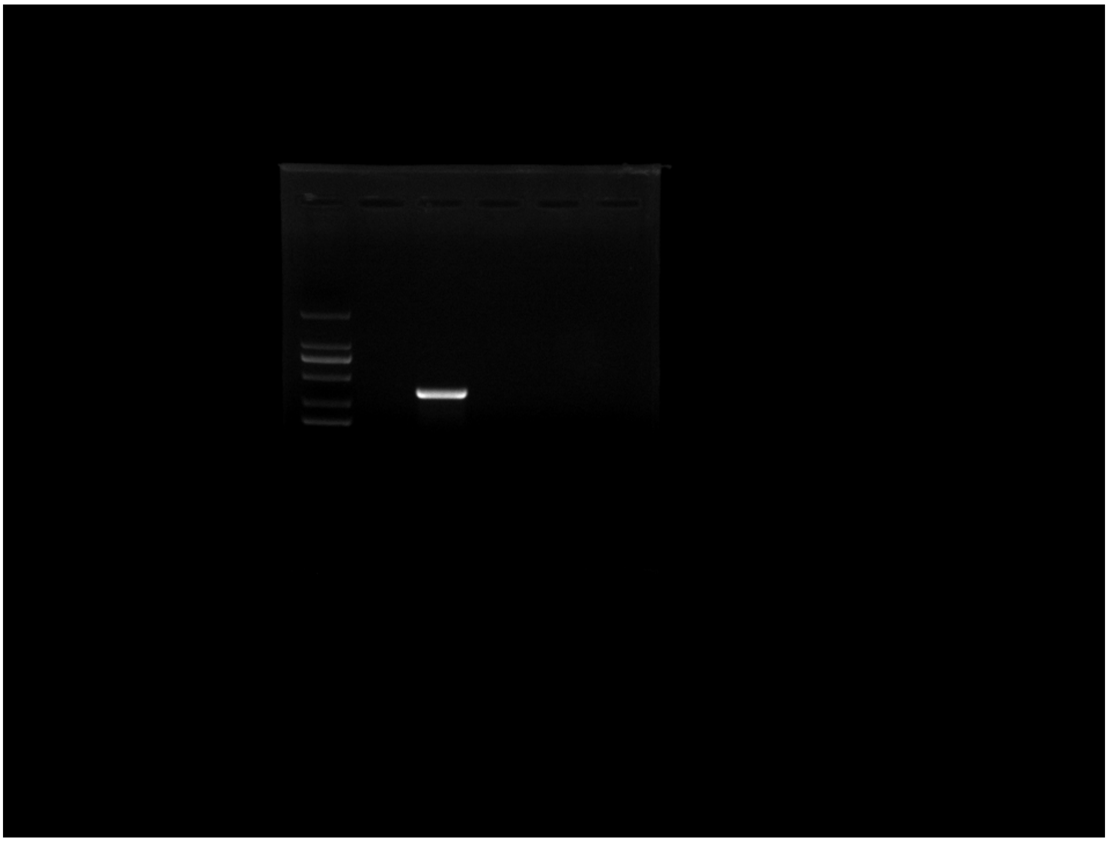

Figure 3. The specificity of nano-PCR. Lane M, DL 2,000 Marker; Lane 1, negative control; Lanes 2-5, FPV, FHV, FCoV and FCV.

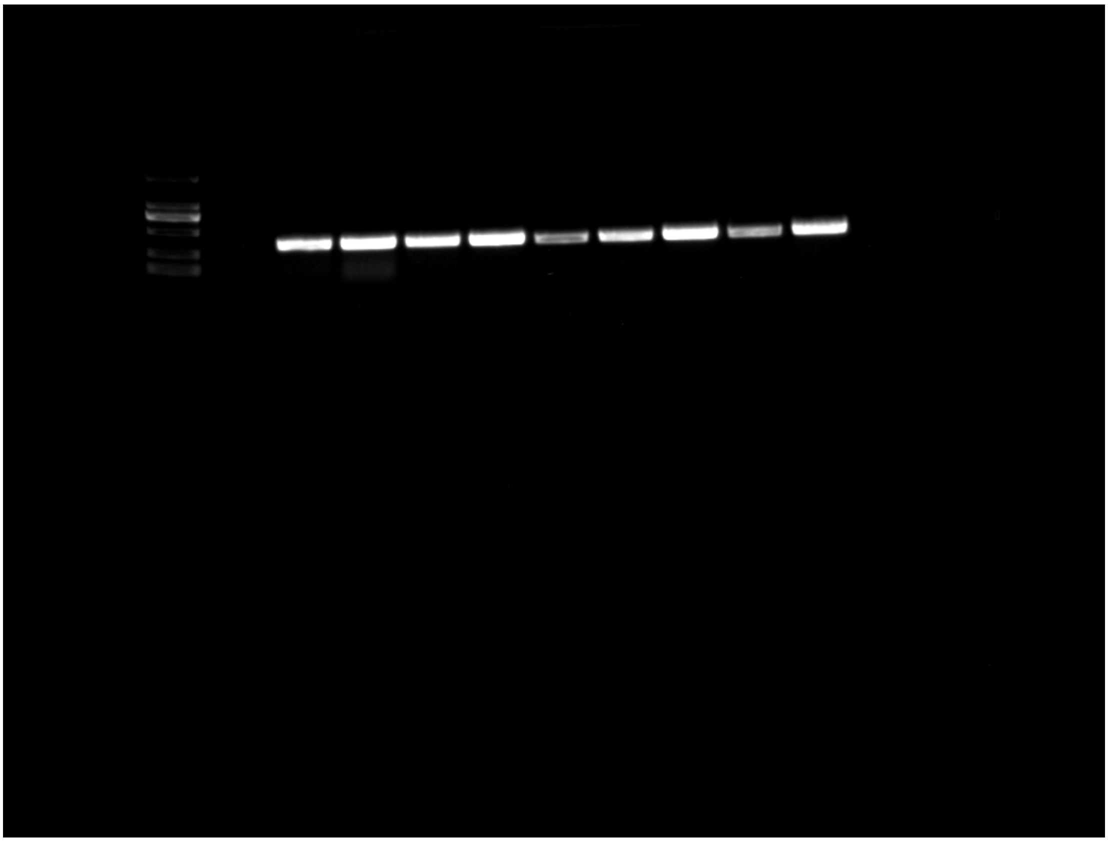

Figure 4. The detection results of some FPV clinical samples. Lane M, DL 2,000 Marker; Lane 1, negative control; Lanes 2-10, FPV clinical samples.
