# Supplementary material for: Development and Application of nanoPCR Method for Detection of Feline Panleukopenia Virus
Source: Vet Sci. 2023 Jul 6;10(7):440. doi: 10.3390/vetsci10070440 (PMC10386105; doi:10.3390/vetsci10070440)
Supplement: Supplementary file 1 [file vetsci-10-00440-s001.zip › Table S1.pdf]

Table. S1: Symptoms and vaccination of 83 samples.

Number	Age (mon)	Clinical symptoms	Vaccine history*	Test strips	Traditional PCR	NanoPCR	Real-time PCR
1	7	Vomiting, hematochezia, fever	+	-**	+***	+	+
2	6	Fever, diarrhea	-	-	-	+	+
3	2	Vomiting, diarrhea	+	-	-	-	-
4	2	Vomiting, fever	-	+	+	+	+
5	7	Dispirited, hematochezia	-	+	+	+	+
6	2	Watery diarrhea	-	+	+	+	+
7	1	Vomiting, watery diarrhea	+	+	+	+	+
8	3	Vomiting, diarrhea	-	+	+	+	+
9	9	Fever, hematochezia	○	+	+	+	+
10	4	Vomiting, diarrhea	-	+	+	+	+

11	7	Dispirited, fever	-	+	+	+	+
12	9	Diarrhea, fever	-	+	+	+	+
13	3	Vomiting, hematochezia	+	+	+	+	+
14	7	Diarrhea, dehydration	+	-	-	-	-
15	4	Watery diarrhea, vomiting	-	-	-	+	+
16	3	Vomiting, sticky feces	○	+	+	+	+
17	7	Vomiting, diarrhea	-	-	-	-	-
18	4	Fever, vomiting	-	+	+	+	+
19	11	Vomiting, diarrhea	-	+	+	+	+
20	2	Fever, diarrhea	○	−	−	+	+
21	9	Fever, vomiting, diarrhea	-	-	+	+	+
22	7	hematochezia	-	+	+	+	+

23	2	Watery diarrhea	-	+	+	+	+
24	3	Vomiting, diarrhea	+	-	-	-	-
25	10	Fever, vomiting, diarrhea	○	+	+	+	+
26	9	Fever, vomiting, dehydration	+	-	-	+	+
27	3	Diarrhea, dehydration	+	-	-	-	-
28	6	Vomiting, hematochezia	○	−	−	+	+
29	4	Vomiting, hematochezia	-	-	-	+	+
30	8	Vomiting, diarrhea	-	+	+	+	+
31	8	Fever, vomiting	-	+	+	+	+
32	9	Vomiting, dispirited	-	+	+	+	+
33	1	Fever, diarrhea	-	-	+	+	+
34	3	Vomiting, diarrhea	-	+	+	+	+

35	4	Diarrhea, dehydration	+	-	-	-	-
36	8	Hematochezia, dispirited	-	+	+	+	+
37	12	Diarrhea	-	-	-	+	+
38	3	Fever, hematochezia	+	-	-	-	-
39	7	Fever, diarrhea	-	+	+	+	+
40	9	Fever, dispirited	-	+	+	+	+
41	8	Fever, vomiting, diarrhea	-	+	+	+	+
42	2	Diarrhea, dehydration	+	+	+	+	+
43	2	Vomiting	-	+	+	+	+
44	3	Hematochezia	-	+	+	+	+
45	2	Watery diarrhea	-	+	+	+	+
46	2	Fever, dispirited	-	-	-	-	-

47	3	Watery diarrhea, vomiting	-	-	-	-	-
48	4	Fever, dispirited	-	+	+	+	+
49	4	Diarrhea	+	-	-	-	-
50	6	Watery diarrhea	+	+	+	+	+
51	4	Fever, Vomiting	-	+	+	+	+
52	7	Vomiting, dispirited	-	-	-	-	-
53	9	Vomiting, diarrhea	○	+	+	+	+
54	14	Diarrhea, dehydration	-	-	-	-	-
55	6	Watery diarrhea	+	-	-	+	+
56	5	Fever, hematochezia	○	-	+	+	+
57	8	Watery diarrhea, vomiting	-	-	-	-	-
58	6	Fever, diarrhea	-	+	+	+	+

59	11	Fever, hematochezia	-	-	-	-	-
60	9	Diarrhea, dehydration	+	+	+	+	+
61	3	hematochezia	+	-	-	+	+
62	6	Fever, diarrhea	-	-	-	-	-
63	7	Watery diarrhea	-	+	+	+	+
64	8	Diarrhea	+	+	+	+	+
65	8	Vomiting, diarrhea	+	+	+	+	+
66	7	Fever, dispirited	-	+	+	+	+
67	6	Fever, diarrhea, dehydration	-	-	-	-	-
68	9	Watery diarrhea	-	+	+	+	+
69	11	Vomiting	-	-	-	-	-
70	2	Fever, dispirited	-	-	-	+	+

71	7	Fever, dispirited	-	-	-	+	+
72	6	Fever, diarrhea	-	+	+	+	+
73	9	Fever, vomiting, diarrhea	-	+	+	+	+
74	6	Fever, hematochezia	-	+	+	+	+
75	3	Fever, hematochezia	+	+	+	+	+
76	4	Fever, vomiting	○	-	-	-	-
77	7	Fever, dispirited	-	+	+	+	+
78	8	Diarrhea	-	+	+	+	+
79	2	Watery diarrhea	+	-	-	-	-
80	6	hematochezia	-	-	-	+	+
81	4	Fever, hematochezia	-	+	+	+	+
82	5	Fever, watery diarrhea	+	+	+	+	+

83	8	Vomiting, diarrhea	-	-	-	+	+

*: Vaccine: Feline Rhinotracheitis-Calici-Panleukopenia Vaccine, Killed Virus; -, unvaccinated vaccine; +, vaccines have been inoculated; ○, incomplete inoculation (incomplete inoculation indicates that at least one or more booster vaccination was not delivered).

**: FPV negative was detected.

***: FPV positive was detected.
